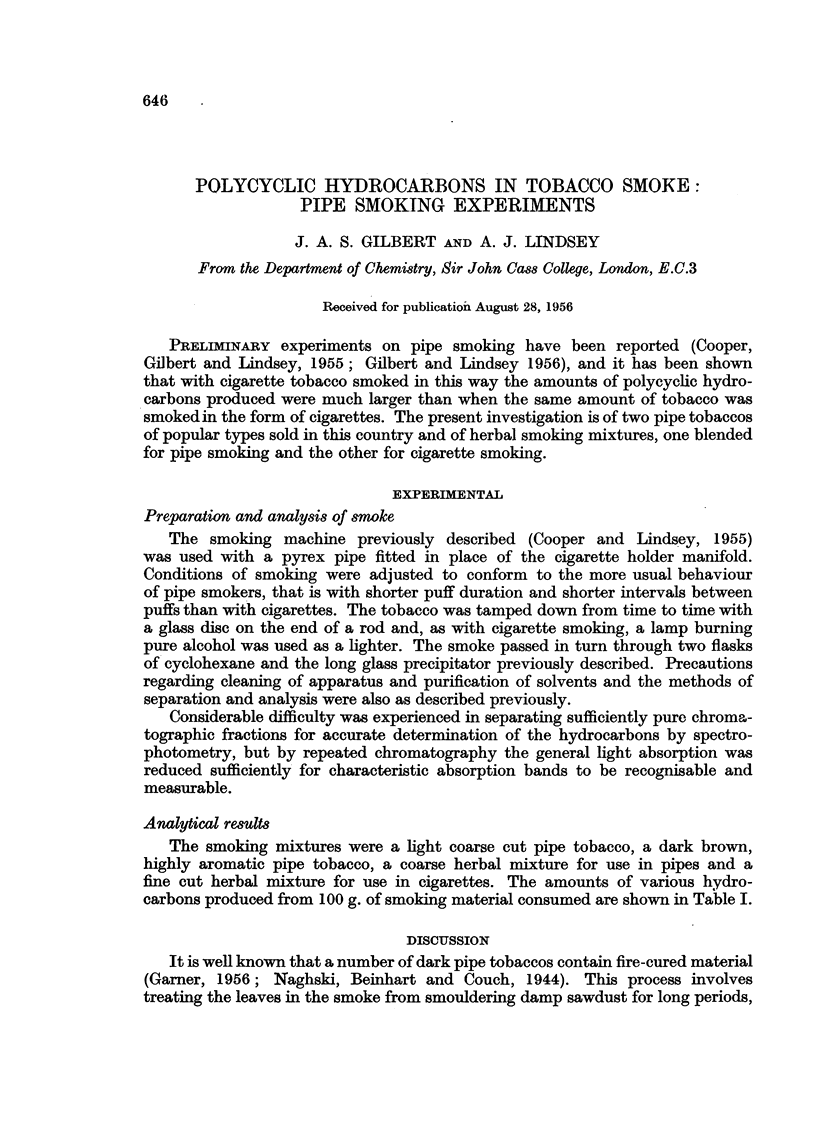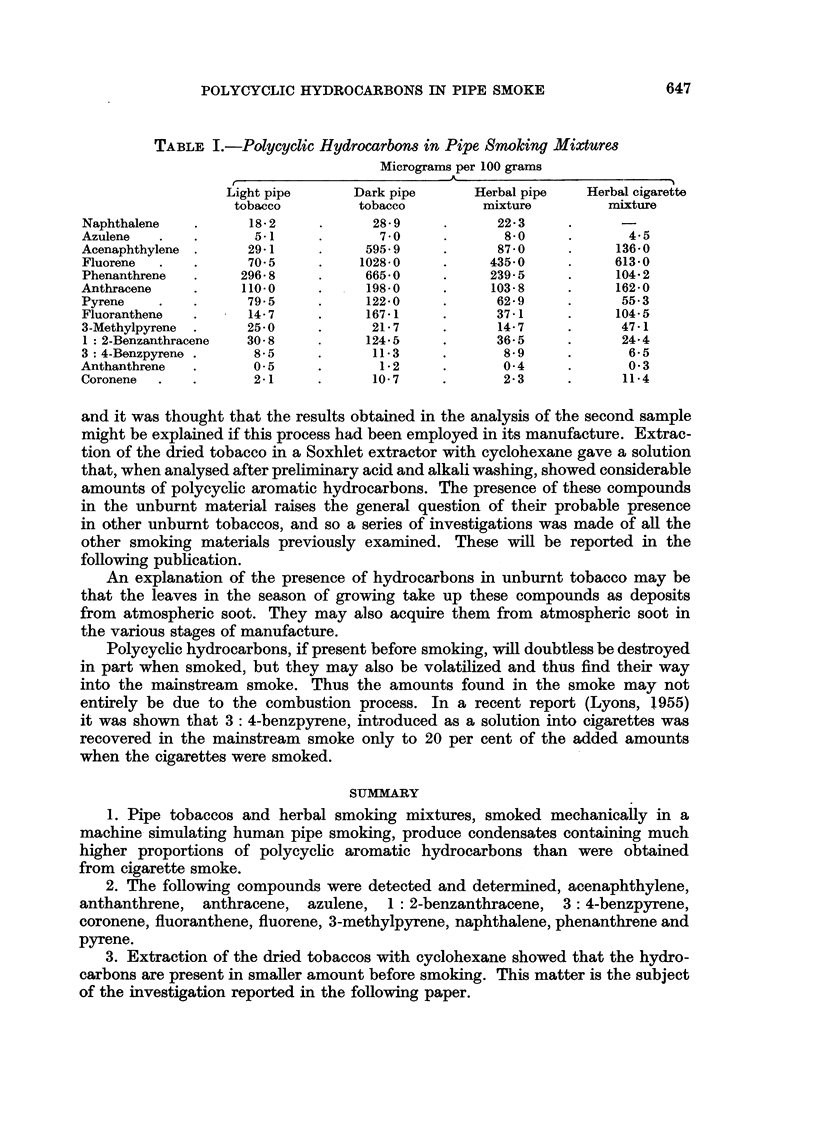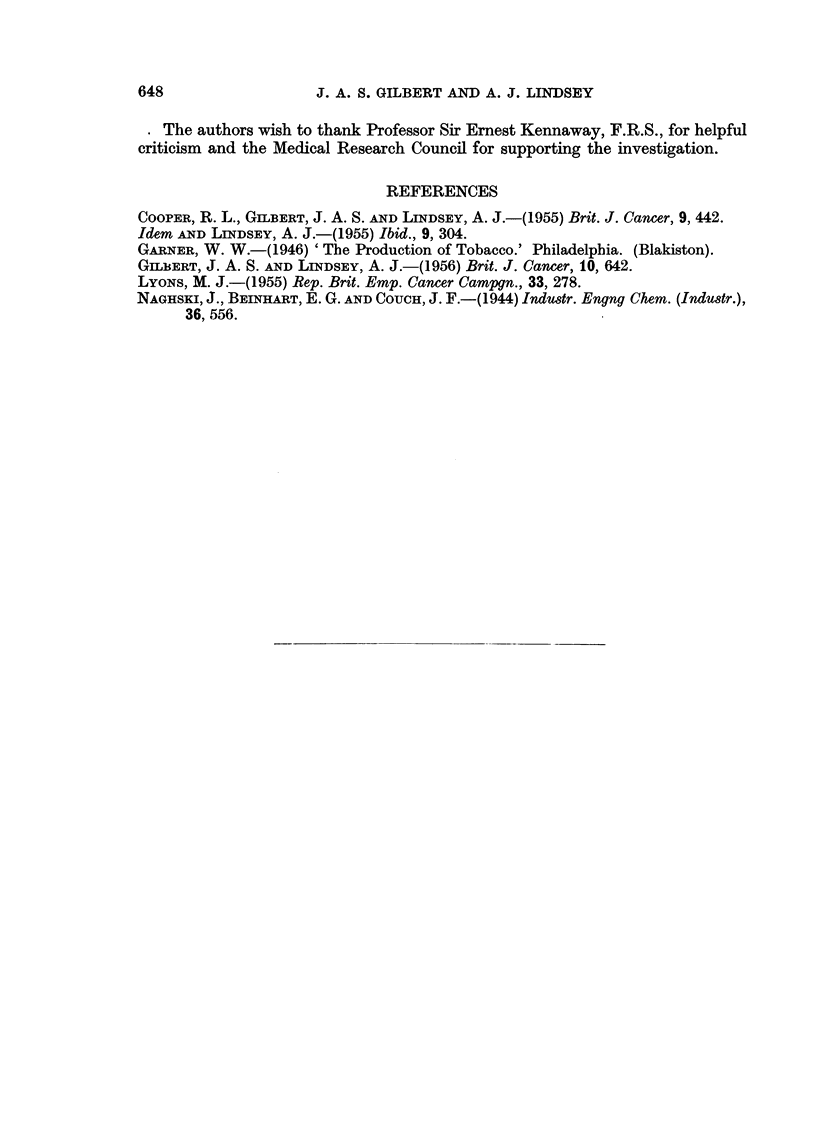# Polycyclic Hydrocarbons in Tobacco Smoke: Pipe Smoking Experiments

**DOI:** 10.1038/bjc.1956.77

**Published:** 1956-12

**Authors:** J. A. S. Gilbert, A. J. Lindsey


### POLYCYCLIC HYDROCARBONS IN TOBACCO SMOKE: PIPE SMOKING EXPERIMENTS

J. A. S. GILBERT AND A. J. LINDSEY

*From the Department of Chemistry, Sir John Cass College, London, E.C.3*

Received for publication August 28, 1956

PRELIMINARY experiments on pipe smoking have been reported (Cooper, Gilbert and Lindsey, 1955; Gilbert and Lindsey 1956), and it has been shown that with cigarette tobacco smoked in this way the amounts of polycyclic hydrocarbons produced were much larger than when the same amount of tobacco was smoked in the form of cigarettes. The present investigation is of two pipe tobaccos of popular types sold in this country and of herbal smoking mixtures, one blended for pipe smoking and the other for cigarette smoking.

### EXPERIMENTAL

### *Preparation and analysis of smoke*

The smoking machine previously described (Cooper and Lindsey, 1955) was used with a pyrex pipe fitted in place of the cigarette holder manifold. Conditions of smoking were adjusted to conform to the more usual behaviour of pipe smokers, that is with shorter puff duration and shorter intervals between puffs than with cigarettes. The tobacco was tamped down from time to time with a glass disc on the end of a rod and, as with cigarette smoking, a lamp burning pure alcohol was used as a lighter. The smoke passed in turn through two flasks of cyclohexane and the long glass precipitator previously described. Precautions regarding cleaning of apparatus and purification of solvents and the methods of separation and analysis were also as described previously.

Considerable difficulty was experienced in separating sufficiently pure chromatographic fractions for accurate determination of the hydrocarbons by spectrophotometry, but by repeated chromatography the general light absorption was reduced sufficiently for characteristic absorption bands to be recognisable and measurable.

### *Analytical results*

The smoking mixtures were a light coarse cut pipe tobacco, a dark brown, highly aromatic pipe tobacco, a coarse herbal mixture for use in pipes and a fine cut herbal mixture for use in cigarettes. The amounts of various hydrocarbons produced from 100 g. of smoking material consumed are shown in Table I.

### DISCUSSION

It is well known that a number of dark pipe tobaccos contain fire-cured material (Garner, 1956; Naghski, Beinhart and Couch, 1944). This process involves treating the leaves in the smoke from smouldering damp sawdust for long periods,

TABLE I.—*Polycyclic Hydrocarbons in Pipe Smoking Mixtures*

| | Micrograms per 100 grams | | | |
|---|---|---|---|---|
| | Light pipe tobacco | Dark pipe tobacco | Herbal pipe mixture | Herbal cigarette mixture |
| Naphthalene | 18·2 | 28·9 | 22·3 | — |
| Azulene | 5·1 | 7·0 | 8·0 | 4·5 |
| Acenaphthylene | 29·1 | 595·9 | 87·0 | 136·0 |
| Fluorene | 70·5 | 1028·0 | 435·0 | 613·0 |
| Phenanthrene | 296·8 | 665·0 | 239·5 | 104·2 |
| Anthracene | 110·0 | 198·0 | 103·8 | 162·0 |
| Pyrene | 79·5 | 122·0 | 62·9 | 55·3 |
| Fluoranthene | 14·7 | 167·1 | 37·1 | 104·5 |
| 3-Methylpyrene | 25·0 | 21·7 | 14·7 | 47·1 |
| 1 : 2-Benzanthracene | 30·8 | 124·5 | 36·5 | 24·4 |
| 3 : 4-Benzpyrene | 8·5 | 11·3 | 8·9 | 6·5 |
| Anthanthrene | 0·5 | 1·2 | 0·4 | 0·3 |
| Coronene | 2·1 | 10·7 | 2·3 | 11·4 |

and it was thought that the results obtained in the analysis of the second sample might be explained if this process had been employed in its manufacture. Extraction of the dried tobacco in a Soxhlet extractor with cyclohexane gave a solution that, when analysed after preliminary acid and alkali washing, showed considerable amounts of polycyclic aromatic hydrocarbons. The presence of these compounds in the unburnt material raises the general question of their probable presence in other unburnt tobaccos, and so a series of investigations was made of all the other smoking materials previously examined. These will be reported in the following publication.

An explanation of the presence of hydrocarbons in unburnt tobacco may be that the leaves in the season of growing take up these compounds as deposits from atmospheric soot. They may also acquire them from atmospheric soot in the various stages of manufacture.

Polycyclic hydrocarbons, if present before smoking, will doubtless be destroyed in part when smoked, but they may also be volatilized and thus find their way into the mainstream smoke. Thus the amounts found in the smoke may not entirely be due to the combustion process. In a recent report (Lyons, 1955) it was shown that 3 : 4-benzpyrene, introduced as a solution into cigarettes was recovered in the mainstream smoke only to 20 per cent of the added amounts when the cigarettes were smoked.

SUMMARY

1. Pipe tobaccos and herbal smoking mixtures, smoked mechanically in a machine simulating human pipe smoking, produce condensates containing much higher proportions of polycyclic aromatic hydrocarbons than were obtained from cigarette smoke.

2. The following compounds were detected and determined, acenaphthylene, anthanthrene, anthracene, azulene, 1 : 2-benzanthracene, 3 : 4-benzpyrene, coronene, fluoranthene, fluorene, 3-methylpyrene, naphthalene, phenanthrene and pyrene.

3. Extraction of the dried tobaccos with cyclohexane showed that the hydrocarbons are present in smaller amount before smoking. This matter is the subject of the investigation reported in the following paper.

The authors wish to thank Professor Sir Ernest Kennaway, F.R.S., for helpful criticism and the Medical Research Council for supporting the investigation.

### REFERENCES

Cooper, R. L., Gilbert, J. A. S. and Lindsey, A. J.—(1955) *Brit. J. Cancer*, **9**, 442.
*Idem* and Lindsey, A. J.—(1955) *Ibid.*, **9**, 304.
Garner, W. W.—(1946) 'The Production of Tobacco.' Philadelphia. (Blakiston).
Gilbert, J. A. S. and Lindsey, A. J.—(1956) *Brit. J. Cancer*, **10**, 642.
Lyons, M. J.—(1955) *Rep. Brit. Emp. Cancer Campgn.*, **33**, 278.
Naghski, J., Beinhart, E. G. and Couch, J. F.—(1944) *Industr. Engng Chem.* (*Industr.*), **36**, 556.